# LncRNA NEAT1 Promotes High Glucose-Induced Mesangial Cell Hypertrophy by Targeting miR-222-3p/CDKN1B Axis

**DOI:** 10.3389/fmolb.2020.627827

**Published:** 2021-01-27

**Authors:** Lin Liao, Jie Chen, Chuanfu Zhang, Yue Guo, Weiwei Liu, Wenrui Liu, Lianxiang Duan, Ziyang Liu, Jing Hu, Jianrao Lu

**Affiliations:** Department of Nephrology, Seventh People’s Hospital of Shanghai University of Traditional Chinese Medicine, Shanghai, China

**Keywords:** NEAT1, Hypertrophy, CDKN1B, ceRNA, miR-222-3p, stat3

## Abstract

Glomerular hypertrophy is an early morphological alteration in diabetic nephropathy. Cyclin-Dependent Kinases have been shown to be required for high glucose (HG)-induced hypertrophy; however, the upstream regulators of CDKN1B in glomerular hypertrophy remain unclear. Herein we describe a novel pathway in which Long noncoding RNA (lncRNA) NEAT1 regulates the progression of mesangial cell hypertrophy via a competing endogenous RNA (ceRNA) mechanism. Real-time PCR was performed to detect the relative NEAT1 and miR-222-3p expressions and further confirmed the relationship between NEAT1 and miR-222-3p. Cell cycle was evaluated by flow cytometry. The related mechanisms were explored by Western blot, RNA immunoprecipitation and chromatin immunoprecipitation assay. We show that NEAT1 forms double stranded RNA (dsRNA) with miR-222-3p, thus limiting miR-222-3p’s binding with CDKN1B. This release of CDKN1B mRNA leads to elevated CDKN1B protein expression, resulting in hypertrophy. In addition, we demonstrated that STAT3 which is activated by HG induces the transcription of NEAT1 by binding to its promoter. Our findings underscore an unexpected role of lncRNAs on gene regulation and introduce a new mode of proliferation regulation in mesangial cells.

## Introduction

Glomerular hypertrophy is a hallmark of hyperglycemia in diabetes mellitus and nephropathy (Hostetter, 2001; Tonneijck et al., 2017). Diabetic nephropathy, a serious and detrimental diagnosis among diabetes patients, accounts for approximately 45% of End Stage Renal Diseases in the United States (Alebiosu and Ayodele, (2006)). Mesangial hypertrophy and cellular expansion are structural phenotypes strongly correlated with diabetic nephropathy (Mauer et al., 1984; Osterby, 1992). Cell-cycle arrest is a commonly observed phenotype among diabetic patients (Vallon and Thomson, 2012), and mesangial cells are known to respond to high-glucose through a phase of proliferation and then arrest in G1 and subsequent hypertrophy (Young et al., 1995). CDK inhibitors, notably CDKN1A and CDKN2B, have been shown to be involved in this hypertrophic non-proliferative state (Wolf, 1998). This phenotype is also observed in mouse knock out models. CDKN1A or CDKN2B deficient mice do not show high glucose (HG)-induced hypertrophy (Monkawa et al., 2002). Although CDK inhibitors have been shown to be involved in regulating glucose-induced hypertrophy, the upstream regulators of CDK inhibitors remains unclear.

Long non-coding RNA (LncRNA) has been increasingly appreciated for having important biological functions. Previously thought to be non-functioning intergenic artifacts, lncRNA is now appreciated as another example of complex genetic organization. The functions of lncRNA are diverse and not fully understood, ranging from imprinting, cis regulation, and antisense interference (Quinn and Chang, 2016). Nuclear enriched abundant transcript 1 (NEAT1) is a highly expressed lncRNA present in the nucleus and is an important player in the formation of paraspeckles (Clemson et al., 2009). NEAT1 has been shown to regulate gene expression by a process called nuclear retention of messenger RNA (mRNA) in paraspeckles (Chen and Carmichael, 2009). NEAT1 is essential for mammary gland development in mice (Standaert et al., 2014). During viral infection, NEAT1 has been shown to stimulate immune response by sequestering IL-8 repressor to paraspeckles (Imamura et al., 2014).

In addition to its role in paraspeckle function, NEAT1 has been shown to be regulate gene expression by acting as competing endogenous RNA (ceRNA) wherein NEAT1 competes with endogenous microRNA (miRNA) and sequester the miRNAs from acting with their mRNA targets (Prinz et al., 2019). NEAT1 has been shown to promote tumorigenesis in several different cancers by regulating the function of several tumor suppressor miRNAs (Prinz et al., 2019). NEAT1 has also been shown to regulate proliferation and fibrosis of mesangial cells, which are specialized cells in the kidney that make up mesangium of glomerulus, in diabetic nephrophathy by activating AKT/mTOR pathway (Huang et al., 2019;Zhang et al., 2019). The physiological consequence of this regulation in cellular hypertrophy remains unclear. Herein, we investigated the role of lncRNA NEAT1 in HG-induced hypertrophy in mesangial cells, and elucidated the underlying molecular mechanism.

## Materials and Methods

### Study Participants

Paraffin-embedded renal tissue sections were collected from patients pathologically diagnosed with diabetic nephropathy by renal biopsy examination that were hospitalized during March 2018 to October 2019 in Seventh People’s Hospital of Shanghai University of Traditional Chinese Medicine. The patients with diabetic nephropathy included 15 men and 15 women with a mean age of 52.4 ± 6.8 year. Paraffin-embedded adjacent normal renal tissue sections obtained from patients with renal carcinoma (15 men and 15 women, with a mean age of 46.8 ± 5.7 year) were taken as controls. Patients who had renal carcinoma with normal kidney function, blood glucose, and urine protein before surgery and without heart or liver disease were included in the study. All participating patients provided signed informed consent before participation in this investigation. Every experimental procedure was approved by the Ethics Committee of Seventh People’s Hospital of Shanghai University of Traditional Chinese Medicine.

### Cell Culture

Human mesangial cells (category No. 4200; ScienCell Research Laboratories, San Diego, California, United States), were cultured in Dulbecco’s modified Eagle’s medium (DMEM) media supplemented with 10% fetal bovine serum (Invitrogen), penicillin (100 U/ml), and streptomycin (100 μg/ml). For the experiments, cells were serum starved for 24 h before supplementing the media with 25 mM glucose (HG) with or without 10 μM AG490 for indicated times. For osmotic control, 5 mM glucose and 20 mM mannitol (NG) were used.

### Gene Knockdown

The NEAT1 shRNA (shNEAT1#1, 5′-GTC​TGT​GTG​GAA​GGA​GGA​A-3′; shNEAT1#2, 5′-TGG​AGG​AGT​CAG​GAG​GAA​T-3′; shNEAT1#3, 5′-GAG​GAG​TCA​GGA​GGA​ATA​G-3′) or scramble shRNA control (shNC, 5′-GTA​GAG​TCA​GCG​AGA​ATC​T-3′) sequences were designed and synthesized by Sangon Biotech (Shanghai, China), and subcloned into pLKO.1 (Addgene, Watertown, MA, United States). To generate the lentivirus, the shRNA and the lentivirus packaging vectors (psPAX2 and pMD2.G, all from Addgene) were transfected into the 293T cells using Lipofectamine 2000 reagent (Invitrogen) following the manufacturer’s protocol. For NEAT1 ectopic expression, the full length NEAT1 was cloned into pLVX-Puro vector (Clontech Laboratories, Inc., Mountain View, CA, United States) using the restriction enzymes *Eco*R I and *Bam*H I, and transfected into the 293T cells as described above. Viruses were collected 48 h post transfection, and were used to infect human mesangial cells. CDKN1B siRNAs or scramble siRNA control (siNC) sequences were designed and synthesized by Sangon Biotech. siRNAs were transfected into the human mesangial cells using Lipofectamine 2000 reagent.

miRNAs were synthesized by Genepharm Technologies (Shanghai, China). Sequences of the miRNAs used in the study are as follows: miR-222-3p mimic (5′-AGC​UAC​AUC​UGG​CUA​CUG​GGU-3′), miR-222-3p inhibitor (5′-ACC​CAG​UAG​CCA​GAU​GUA​GCU-3′), and negative control (NC, 5′-CAG​UAC​UUU​UGU​GUA​GUA​CAA-3′). miRNAs were transfected using Lipofectamine 2000 reagent.

### Cell Cycle Assay

Cells transduced with indicated vectors were collected by centrifugation at 1000 x g for 5 min. The cells were then fixed with pre-cooled absolute ethyl alcohol and incubated with 1 mg/mL of RNase A in dark for 30 min. After that, the cells were stained with 50 μg/mL of propidium iodide (PI) for 10 min, and the cell cycle state was examined with Accuri™ C6 flow cytometer (BD Biosciences).

### Protein Synthesis

Cells grown in media containing HG were incubated with [^35^S]-methionine, and incorporation of [^35^S]-methionine was quantified as described previously (Dey et al., 2011) to measure the protein synthesis.

### Cellular Hypertrophy

Cells were trypsinized and counted in a hemocytometer. The cells were then centrifuged at 4000 ×g at 4°C, and were lysed in RIPA buffer after washing 1X with PBS, and the protein content was measured using the bicinchoninic acid (BCA) Protein Assay Kit (Beyotime). Hypertrophy was determined as described previously (Dey et al., 2011) as the ratio of total cellular protein to the cell number.

### Luciferase Reporter Assay

NEAT1 containing miR-26a-5p complementary sequence was cloned into pGL3-Promoter firefly luciferase reporter vector (Promega). For NEAT1 luciferase reporter assay, cells were transfected with miR-222-3p mimic or miR-222-3p inhibitor and pGL3-Promoter-NEAT1-WT (NEAT1-WT) or pGL3-Promoter-NEAT1-MUT (NEAT1-MUT) and pRL-TK vector (Promega) expressing the renilla luciferase using Lipofectamine 2000 reagent. For the CDKN1B luciferase reporter assay, CDKN1B 3′-UTR sequence was cloned into the pGL3-Promoter vector. Human mesangial cells transduced with miR-222-3p mimic or miR-222-3p inhibitor were co-transfected with pGL3-Promoter-CDKN1B 3′-UTR-WT (CDKN1B-WT) or pGL3-Promoter-CDKN1B 3′-UTR-MUT (CDKN1B-MUT) and pRL-TK vector. For NEAT1 promoter activity, full length NEAT1 promoter was inserted into pGL3-Enhancer firefly luciferase reporter vector (Promega). Human mesangial cells treated with NG or HG in the absence or presence of 10 μM JAK2/STAT3 signaling inhibitor AG490 (MedChemExpress, Monmouth Junction, NJ, United States) were co-transfected with pGL3-Enhancer-NEAT1 promoter and pRL-TK vector. The restriction enzymes *Kpn* I and *Nhe* I were used for these reporter constructs. Cells were harvested to measure firefly and *Renilla* luciferase activities by the dual-luciferase assay kit (Promega) after 48 h of cotransfection. The relative luciferase activity was normalized by the ratio of Firefly and Renilla luciferase activities.

### Real-Time PCR

RNA was extracted using the TRIzol reagent (Invitrogen, Carlsbad, CA, United States) and reverse-transcribed into cDNA with Oligo(dt)18 primer (Thermo Fisher Scientific, Rockford, IL, United States) with cDNA synthesis kit (Thermo Fisher Scientific). Real-time PCR was performed using Maxima SYBR Green qPCR Master Mixes (Thermo Fisher Scientific) on an ABI 7900 System (Applied Biosystem, Foster City, CA, United States) according to the manufacturer’s instructions, with NEAT1, CDKN1B or GAPDH-specific forward primer (NEAT1 forward 5’-CCT​CCC​TTT​AAC​TTA​TCC​ATT​C-3′ and reverse 5′-TCC​ACC​ATT​ACC​AAC​AAT​AC-3′; CDKN1B forward 5′-TGT​CCA​TTT​ATC​CAC​AGG​AAA​G-3′ and reverse 5′-TTC​TAC​CCA​ACA​CAG​CAT​TTA​C-3′; GAPDH forward 5′-AAT​CCC​ATC​ACC​ATC​TTC-3′ and reverse 5′-AGG​CTG​TTG​TCA​TAC​TTC-3′). GAPDH was used for the normalization. Stem-loop real-time RT-PCR was carried out to analyze miRNA expression. U6 RNA was used as a miRNA internal control. Briefly, extracted RNAs were converted into cDNAs with miR-222-3p reverse transcription primer (5′-GTC​GTA​TCC​AGT​GCA​GGG​TCC​GAG​GTA​TTC​GCA​CTG​GAT​ACG​ACA​CCC​AG-3’) and Oligo(dt)18 primer with cDNA synthesis kit (Thermo Fisher Scientific). Real-time PCR was then performed using Maxima SYBR Green qPCR Master Mixes (Thermo Fisher Scientific) according to the manufacturer’s instructions, with miR-222-3p or U6-specific forward primer (miR-222-3p forward 5′-GCG​CGA​GCT​ACA​TCT​GGC​TA-3′ and reverse 5′-AGT​GCA​GGG​TCC​GAG​GTA​TT-3′; U6 forward 5′-CTC​GCT​TCG​GCA​GCA​CA-3′ and reverse 5′-AAC​GCT​TCA​CGA​ATT​TGC​GT-3′). The 2^−ΔΔCt^ method was used to calculate the relative expression.

### Western Blotting

The cells were lysed using RIPA buffer (Beyotime), and protein concentration was measured using the bicinchoninic acid (BCA) Protein Assay Kit (Beyotime). In each lane, 20 μg of protein was loaded, and proteins were separated by 10% SDS-PAGE gels and electroblotted onto polyvinylidene fluoride (PVDF) membranes (Millipore). The membrane was blocked with 5% fat-free milk for 1 h at room temperature, and probed with primary antibodies against CDKN1B (Abcam, ab32034, 1:5000) and GAPDH (Cell Signaling Technology, #5174, 1:2000) overnight at 4°C. The membrane was washed 3X with Tris-Buffered Saline Tween-20 (TBST), and then incubated with the horseradish peroxidase (HRP)-conjugated secondary antibodies (Beyotime, A0208 and A0216, 1:1000). Blots were examined by an Enhanced Chemiluminescence (ECL) Detection kit (Pierce Biotechnology). The band intensity was quantified with Image-Pro Plus 6.0 software.

### Fluorescence in Situ Hybridization

The FISH assay was performed in human mesangial cells using a FISH kit (RiboBio, Guangzhou, China) following the manufacturer’s instructions. After permeabilization, the cells were incubated with lncRNA NEAT1 probe at 37°C overnight. The cell nuclei were stained with DAPI (Sigma-Aldrich). The staining results were observed using a fluorescence microscope (Nikon, Japan).

### RNA Immunoprecipitation (RIP) Assays

RNAs were immunoprecipitated (IP) using Magna RIP RNA-Binding Protein Immunoprecipitation kit (Millipore) following the manufacturer’s instructions. Briefly, human mesangial cells were lysed in RIP lysis buffer, and RNAs magnetic beads were conjugated with anti-AGO2 (Abcam, ab186733, 1:30) or anti-IgG antibody (Abcam, ab172730, 1:30) overnight at 4°C and washed with RIP-wash buffer for 10 min at 4°C and then RIP-lysis buffer for 5 min at 4°C. The coprecipitated RNAs were used for cDNA synthesis and evaluated by Real-time PCR as described above.

### RNA Pull-Down Assay

The bio-labeled probe of miR-222-3p (Bio-miR-222-3p) and blank control (Bio-NC) were synthesized by Sangon (Shanghai, China). Then Bio-miR-222-3p or Bio-NC was transfected into human mesangial cells. Subsequently, cells were lysed and incubated with Streptavidin-Dyna beads overnight at 4°C along with RNA separation. Finally, the enrichment of NEAT1 was identified by Real-time PCR.

### Chromatin Immunoprecipitation

ChIP was performed as previously described (Zhu et al., 2017). Crosslinked chromatin was immunoprecipitated with anti-STAT3 (Cell Signaling Technology, #4904, 1:1000) or anti-IgG antibody (Abcam, ab172730, 1:30). Binding was detected by Real-time PCR with NEAT1 promoter primer (Forward, 5′-AGG​GGT​CTT​CTT​CCT​CAT​GG-3′ and Reverse, 5′-TGC​TCA​ACG​GGA​CGA​TTC-3′).

### Statistical Analysis

GraphPad Prism 8.0.2 (GraphPad Software) was used for statistical analysis. Three independent experiments were performed for each data point, and the data represent the mean ± standard deviation (SD) of the triplicates. For experiments with only two groups, two-sided Student’s t test was used for comparisons of group means. For experiments with more than two groups, the differences between groups were compared by ANOVA followed by Dunnett’s test, in which all groups were tested with the control group, or Tukey’s test, in which all groups were tested with each other. P values <0.05 were considered statistically significant.

## Results

### NEAT1 Promotes HG-Induced Hypertrophy

**FIGURE 1:**
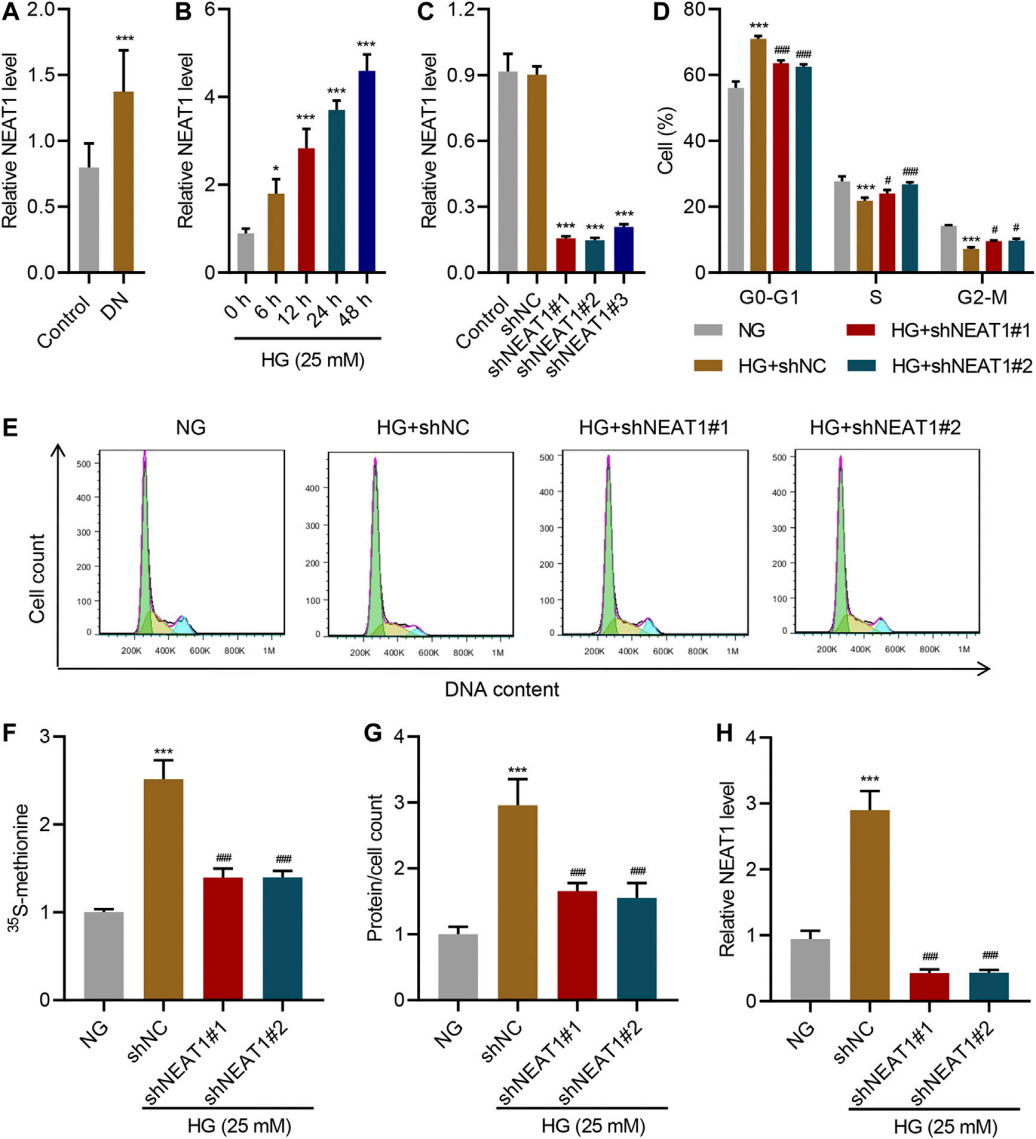
NEAT1 promotes HG-induced hypertrophy. **(A)** Expression of NEAT1 in renal tissues of patients with diabetic nephropathy (DN, n = 30) versus controls (n = 30) determined by Real-time PCR. Human mesangial cells were treated with 5 mM glucose and 20 mM mannitol (NG) or 25 mM glucose (HG); **(B)** NEAT1 expression was measured by Real-time PCR in cells treated with HG for indicated times. (C-H) Cells were transfected with control vector or NEAT1 shRNA, and cells were treated with NG or HG for 24 h; **(C)** Expression of NEAT1 was measured by Real-time PCR; **(D, E)** Distribution of cells in different stages of cell cycle was quantified by FACS; **(F)** Protein synthesis was measured by quantifying the incorporation of ^35^S-methionine; **(G)** Hypertrophy was measured by quantifying average protein content per cell; **(H)** Expression of NEAT1 was measured by Real-time PCR. * indicates *P*<0.05, *** indicates *P*<0.001 compared with control or NG, ^#^ indicates P<0.05, ^###^ indicates *P*<0.001 compared with HG + shNC. Data were expressed as mean ± SD, and experiments were repeated three times.

To confirm the dysregulation of NEAT1 in diabetic nephropathy, Real-time PCR was conducted to determine NEAT1 expression in clinical renal tissues obtained from patients with diabetic nephropathy. The Real-time PCR data showed that NEAT1 expression in patients with diabetic nephropathy was significantly higher than that in controls (Figure 1A). To test the potential role of NEAT1 in glucose-induced hypertrophy, human mesangial cells were cultured in HG, and expression of NEAT1 was measured by Real-time PCR. NEAT1 expression increased in a time dependent manner upon treatment with HG (Figure 1B). To test the functional role of NEAT1 in glucose-induced hypertrophy, NEAT1 was knocked down using short hairpin RNA (shRNA) targeting NEAT1 (Figure 1C), and change in cell cycle was examined. Indeed, NEAT1 knock down showed significantly fewer cells in G0-G1, and significantly higher more cells in S and G2/M phases (Figure 1D E).

Next, we measured the protein level upon HG treatment and NEAT1 knock down. HG treatment increased the amount of newly synthesized proteins as measured by the incorporation of ^15^S-methionine, and ^15^S-methionine incorporation was significantly reduced in NEAT1 knock down cells (Figure 1F). Similar result was observed in hypertrophy, evidenced by the increased total protein level per cell (Figure 1G), and on the RNA level of NEAT1 (Figure 1H).

**FIGURE 2:**
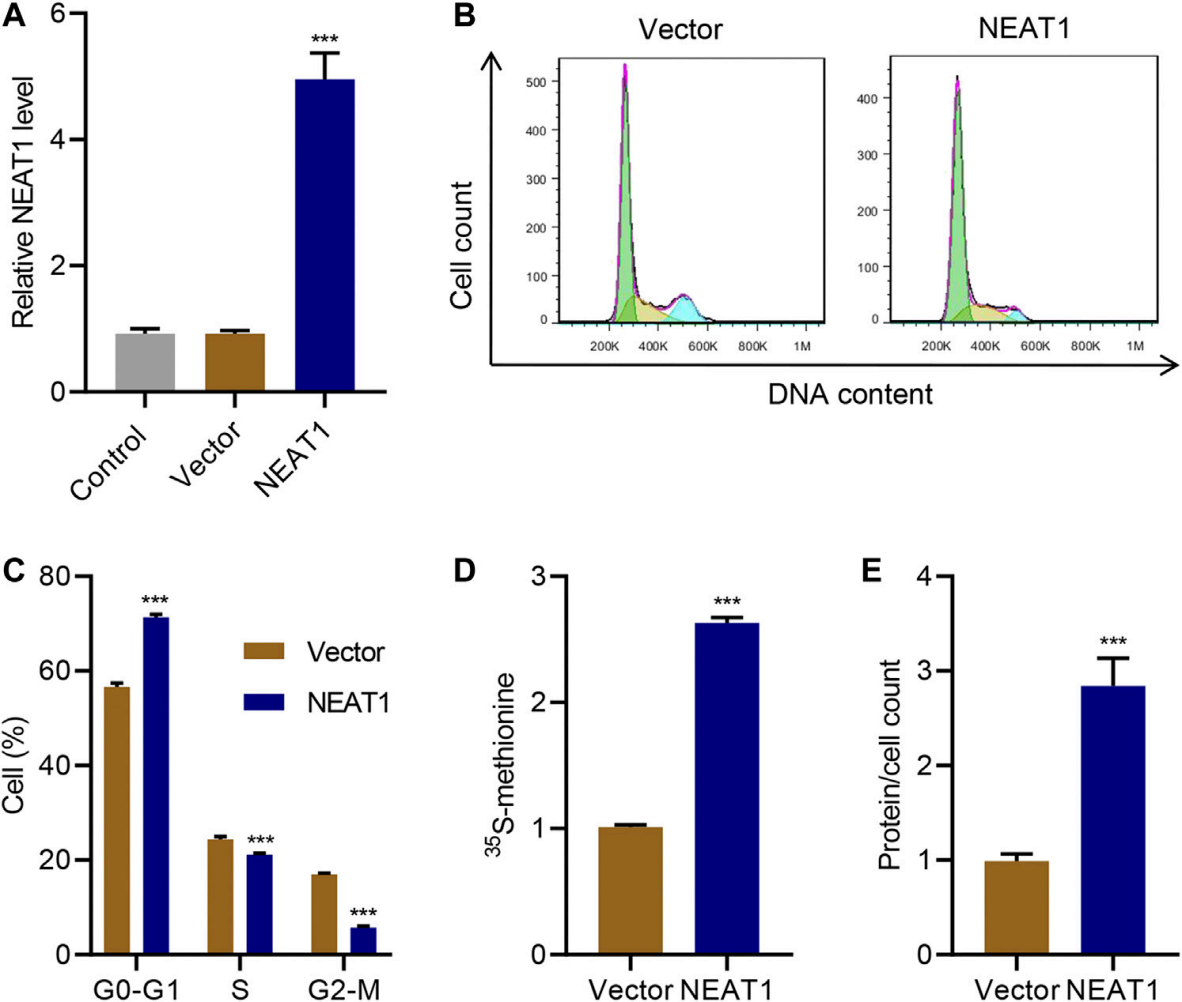
NEAT1 overexpression promotes mesangial cell hypertrophy. Human mesangial cells were transduced with NEAT1 expressing lentivirus or control vector. **(A)** NEAT1 expression was measured by Real-time PCR. The effect of NEAT1 overexpression on; **(B, C)** cell cycle was measured by PI incorporation; **(D)** protein synthesis was measured by ^35^S-methionine incorporation, and; **(E)** hypertrophy was measured by quantifying average protein content per cells. *** indicates *P*<0.001 compared with vector. Data were expressed as mean ± SD, and experiments were repeated three times.

On the other hand, when NEAT1 was ectopically overexpressed (Figure 2A), cells in G0-G1 was significantly increased, and cells in S and G2/M phase was significantly decreased (Figure 2B C). Likewise, the new protein synthesis was significantly increased upon NEAT1 overexpression (Figure 2D), and total proteins per cells was significantly higher in the cells overexpressing NEAT1 (Figure 2E). Collectively, these results show that NEAT1 promotes HG-induced hypertrophy.

### NEAT1 Promotes Hypertrophy by Negatively Regulating miR-222-3p Expression

**FIGURE 3:**
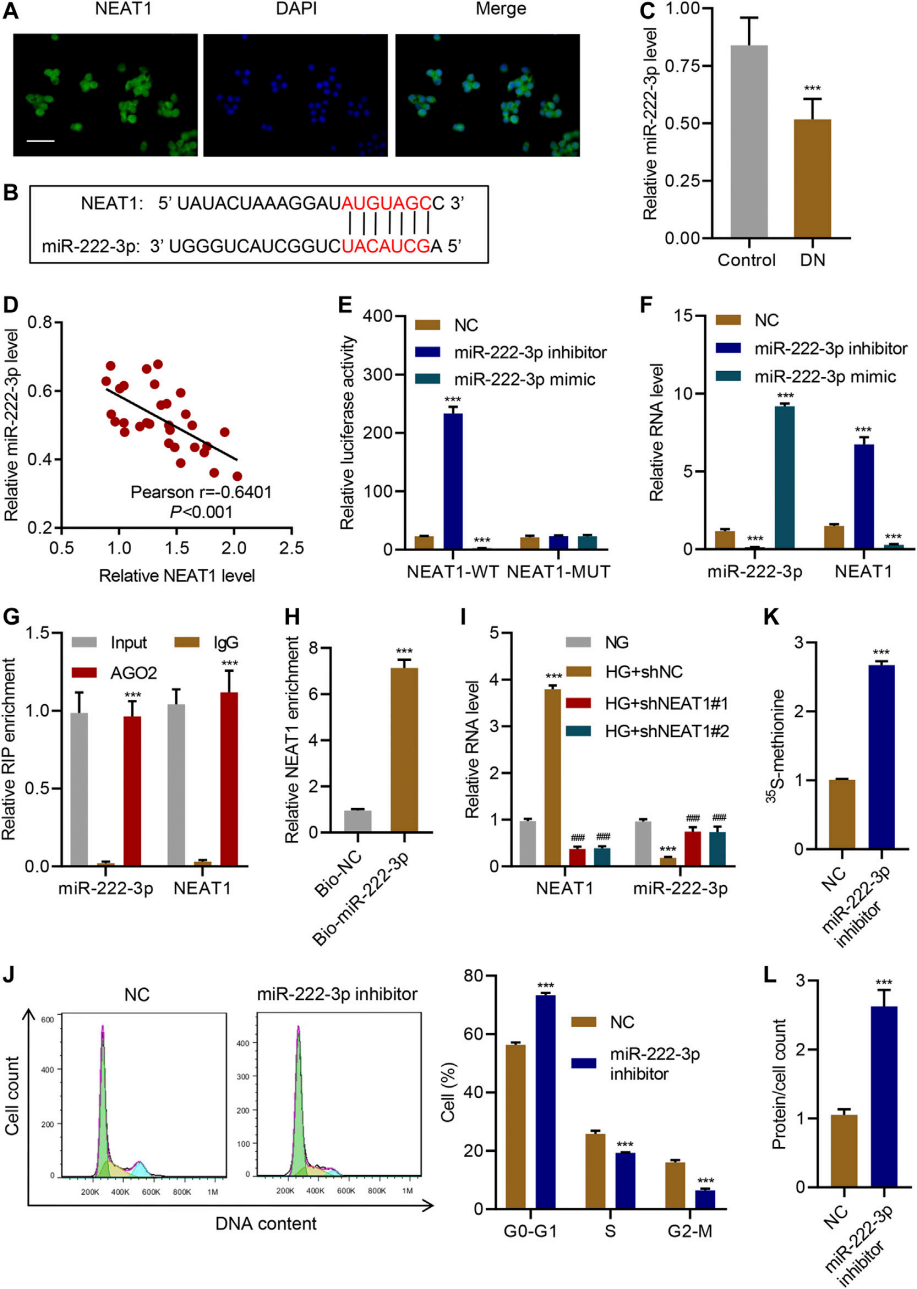
NEAT1 promotes hypertrophy by negatively regulating miR-222-3p expression. **(A)** The location of NEAT1 in human mesangial cells was determined by FISH assay. DAPI-stained nuclei are blue. Scale bar, 50 μm; **(B)** The sequence of miR-222-3p and NEAT1 showing the potential binding sites between the two RNAs; (**C**) Expression of miR-222-3p in renal tissues of patients with diabetic nephropathy (DN, n = 30) versus controls (n = 30) determined by Real-time PCR; **(D)** Pearson correlation; scatter plots in renal tissues of patients with diabetic nephropathy (n = 30). (E, F) Human mesangial cells were co-transfected with luciferase gene with wild-type NEAT1 (NEAT1-WT) or mutant NEAT1 (NEAT1-MUT), and miR-222-3p mimic, miR-222-3p inhibitor, or NC, and; **(E)** luciferase activity was measured by luciferase reporter assay and; **(F)** indicated RNAs were measured by Real-time PCR; **(G)** Anti-AGO2 was used to immunoprecipitate AGO2, and binding of NEAT1 or miR-222-3p was measured by Real-time PCR; **(H)** RNA pull-down assay was performed to verify the relationship between miR-222-3p and NEAT1; **(I)** miR-222-3p and NEAT1 expression in human mesangial cells infected with indicated lentiviral vectors and treated with NG or HG for 24 h was measured by Real-time PCR; **(J)** Cell cycle; **(K)** protein synthesis and; **(L)** hypertrophy in human mesangial cells transfected with miR-222-3p inhibitor or NC were measured. *** indicates *P*<0.001 compared with control, NC, IgG, Bio-NC or NG. Data were expressed as mean ± SD, and experiments were repeated three times.

Next, we sought to identify the molecular mechanism through which NEAT1 regulates hypertrophy. FISH analysis confirmed that NEAT1 was mainly located in the cytoplasm (Figure 3A). Examination of NEAT1 sequence revealed that a portion of the sequence is complementary to the sequence of miR-222-3p (Figure 3B), whose expression was decreased upon HG stimulation and in diabetic rats (Li et al., 2015). The Real-time PCR data showed that miR-222-3p expression in patients with diabetic nephropathy was significantly lower than that in controls (Figure 3C), and a negative correlation between NEAT1 and miR-222-3p expression was observed in diabetic nephropathy patients (Figure 3D). NEAT1 sequence containing miR-222-3p complementary sequence (WT) or the sequence with mutation in the complementary sequence (MUT) was cloned downstream of luciferase reporter, and activity of the luciferase in cells expressing miR-222-3p inhibitor or miR-222-3p mimic was measured. In WT, miR-222-3p inhibitor caused an increase in the activity of the luciferase whereas the miR-222-3p mimic caused a significant reduction in the activity of the luciferase (Figure 3E). On the other hand, either the inhibitor or the mimic had no effect in the expression of the luciferase in MUT cells (Figure 3E). As expected, miR-222-3p inhibitor also significantly increased the level of NEAT1 and miR-222-3p mimic significantly reduced the expression of NEAT1 (Figure 3F).

To further verify the direct role of miRNA in regulating the expression of NEAT1, we immunoprecipitated AGO2, which forms the catalytic subunit of RNA induced silencing complex (RISC), and checked the binding of NEAT1 and miR-222-3p by RIP. Indeed, NEAT1 as well as miR-222-3p were found to interact with AGO2 (Figure 3G). In addition, RNA pull-down assays was also clarified the interaction between NEAT1 and miR-222-3p (Figure 3H). These results revealed that miR-222-3p was directly targeted by NEAT1.

**FIGURE 4:**
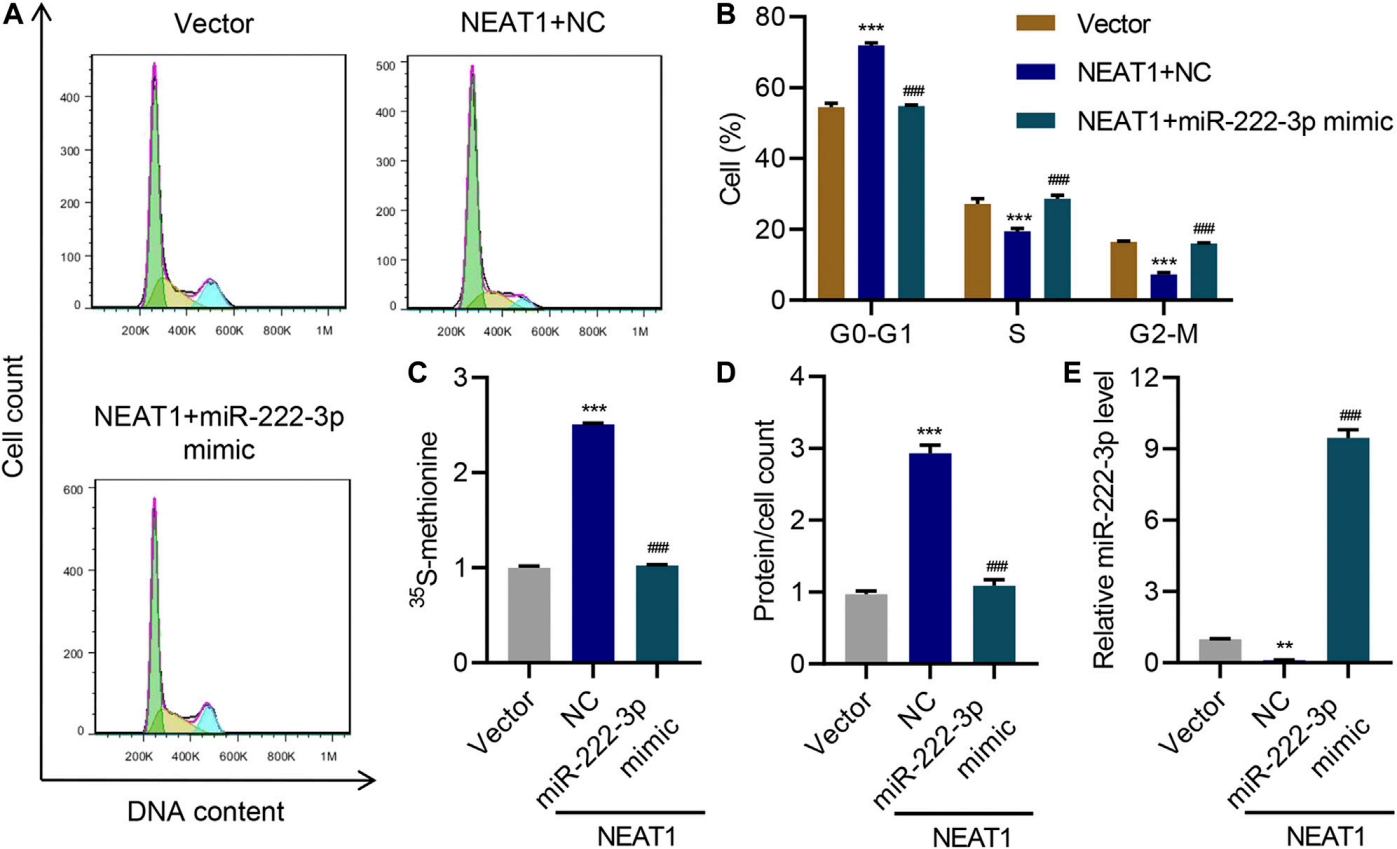
miR-222-3p antagonizes the effect of NEAT1 on hypertrophy. Human mesangial cells were infected with empty vector or lentivirus expressing NEAT1, and transfected with NC or miR-222-3p mimic, and the effect on **(A, B)** cell cycle; **(C)** protein synthesis; **(D)** hypertrophy and; **(E)** miR-222-3p expression was measured. ** indicates *P*<0.01, *** indicates *P*<0.001 compared with vector. ^###^ indicates *P*<0.001 compared with NEAT1 + NC. Data were expressed as mean ± SD, and experiments were repeated three times.

Interestingly, HG had opposite effect on the expression of miR-222-3p compared to NEAT1. HG caused a decrease in the expression of miR-222-3p whereas NEAT1 knock down caused an increase in the expression of miR-222-3p (Figure 3I). Functionally, miR-222-3p inhibitor caused significant increase in G0-G1 subpopulation and significant decrease in S and G2/M subpopulation (Figure 3J). Likewise, the ^15^S-methionine incorporation and total proteins per cells were significantly higher in miR-222-3p inhibitor treated cells (Figure 3K L). In contrast, expression of exogenous expression of NEAT1 together with miR-222-3p blunted the effect of NEAT1 on cell cycle regulation, protein synthesis and protein content per cell (Figure 4A–E). Collectively, these results indicate that HG promotes hypertrophy in mesangial cells by downregulating the expression of miR-222-3p that targets NEAT1 for degradation.

### NEAT1 Regulates CDKN1B Expression Through miR-222-3p

**FIGURE 5:**
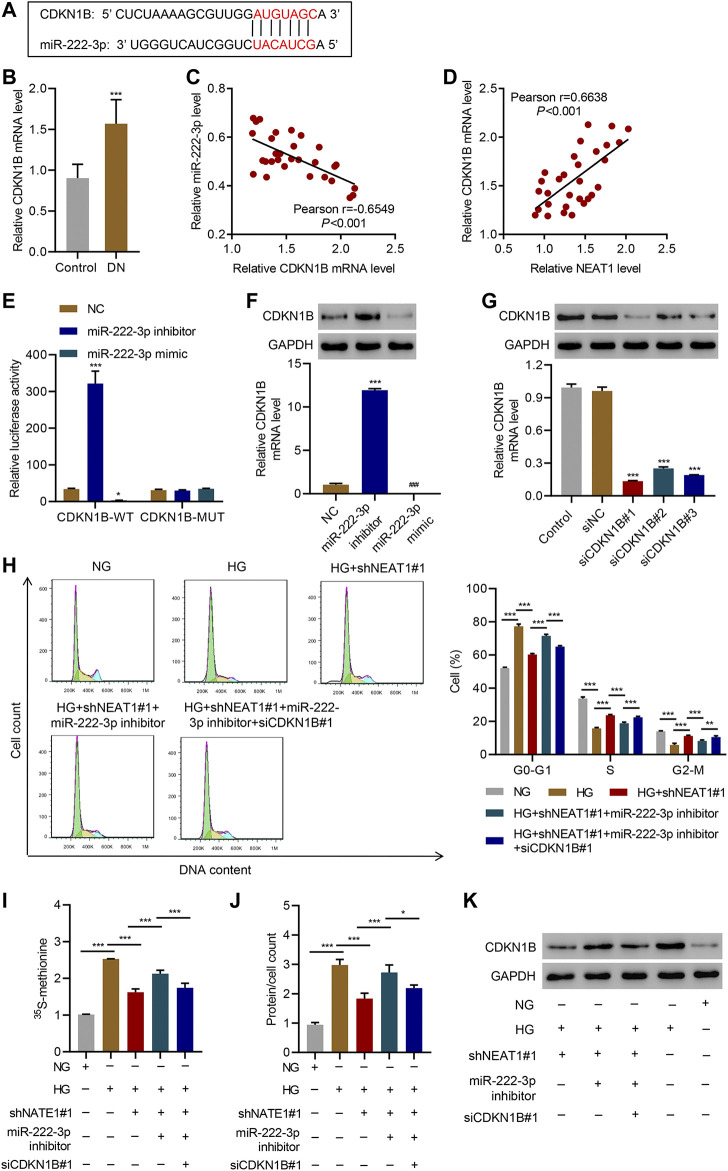
NEAT1 regulates CDKN1B expression by segregating miR-222-3p. **(A)** The potential binding sequence between miR-222-3p and CDKN1B are shown; **(B)** Expression of CDKN1B in renal tissues of patients with diabetic nephropathy (DN, n = 30) versus controls (n = 30) determined by Real-time PCR; **(C, D)** Pearson correlation scatter plots in renal tissues of patients with diabetic nephropathy (n = 30); **(E)** Luciferase assays in human mesangial cells co-transfected with luciferase gene with wild-type (CDKN1B-WT) or mutant CDKN1B (CDKN1B-MUT) and miR-222-3p mimic, miR-222-3p inhibitor, or NC were performed; **(F-K)** Human mesangial cells were treated with either NG or HG for 24 h, transfected with miR-222-3p mimic, miR-222-3p inhibitor, or NC, or infected with indicated lentiviral vectors; **(F, G)** CDKN1B expression was measured by WB and Real-time PCR; **(H)** Cell cycle; **(I)** protein synthesis, **(J)** hypertrophy and; **(K)** CDKN1B expression was measured. * indicates; *P*<0.05, *** indicates *P*<0.001 compared with control, NC or siNC (B, E-G). * indicates *P*<0.05, ** indicates *P*<0.01, *** indicates *P*<0.001 (I-J). Data were expressed as mean ± SD, and experiments were repeated three times.

miRNAs normally regulate the expression of protein coding genes by directly targeting their 3’ UTR, hence we investigated if miR-222-3p also targets any protein coding genes in addition to NEAT1. We found that miR-222-3p sequence is complementary to the 3’ UTR of CDKN1B (Figure 5A), whose expression was increased in glomeruli of diabetic animals and mesangial cells cultured under HG (Awazu et al., 2003) and associated with HG-induced mesangial cell hypertrophy (Wang et al., 2015). The Real-time PCR data showed that CDKN1B expression in patients with diabetic nephropathy was significantly higher than that in controls (Figure 5B), and a negative correlation between CDKN1B and miR-222-3p expression (Figure 5C) while a positive correlation between CDKN1B and NEAT1 expression was observed in diabetic nephropathy patients (Figure 5D). To test if miR-222-3p regulates the expression of CDKN1B, we expressed miR-222-3p inhibitor or mimic, and examined the effect on the expression of CDKN1B. Indeed, the expression of miR-222-3p inhibitor increased the expression of luciferase reporter with WT 3’ UTR of CDKN1B, but had no effect in the expression of luciferase with mutant 3’ UTR of CDKN1B that lacked the complementary sequence of miR-222-3p (Figure 5E). In contrast, the miR-222-3p mimic had opposite effect on the expression of luciferase with WT 3’ UTR of CDKN1B, and had no effect in the expression of luciferase with mutant 3’ UTR of CDKN1B (Figure 5E). As expected, the miR-222-3p inhibitor caused an increase in the mRNA and protein level of endogenous CDKN1B (Figure 5F), and miR-222-3p mimic reduced the mRNA and protein level of endogenous CDKN1B (Figure 5F).

Next, we investigated if NEAT1 regulates the expression of CDKN1B through miR-222-3p. We knocked down CDKN1B (Figure 5G), and examined the effect of NEAT1 knock down and miR-222-3p expression in cell cycle, protein synthesis and protein content. Indeed, CDKN1B knock down reversed the effect of NEAT1 knock down and miR-222-3p inhibitor on cell cycle profile, protein synthesis and protein content (Figure 5H–J). Furthermore, the expression of CDKN1B was reduced with NEAT1 knock down and was reversed with miR-222-3p treatment (Figure 5K). Collectively, these results demonstrate that NEAT1 mitigates the effect of miR-222-3p on CDKN1B, and thus regulates hypertrophy in mesangial cells.

### STAT3 Regulates the Transcription of NEAT1

**FIGURE 6:**
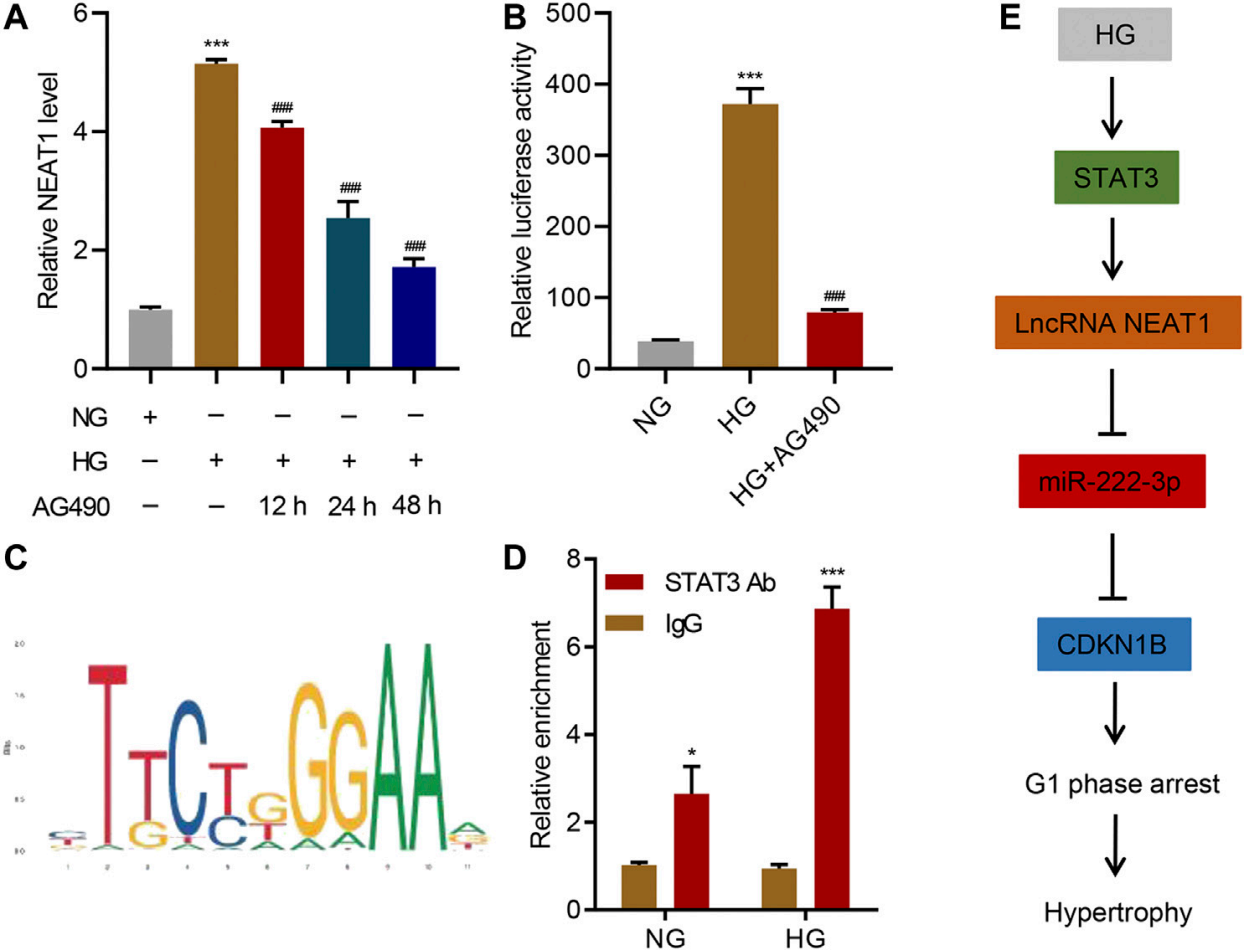
STAT3 regulates the transcription of NEAT1. **(A)** LncRNA NEAT1 expression in human mesangial cells treated with NG or HG in the absence or presence of 10 μM AG490 for indicated times was measured by Real-time PCR; **(B)** Luciferase assays were performed using the promoter of NEAT1 in human mesangial cells treated with NG, HG, and HG plus 10 μM AG490 for 24 h; **(C)** The binding motif of STAT3 in the promoter of NEAT1 was identified using the JASPAR database; **(D)** ChIP assays with anti-STAT3 antibody were performed in human mesangial cells treated with NG or HG, and binding of STAT3 in the promoter of NEAT1 was measured by Real-time PCR; **(E)** Proposed model of the regulation of STAT3/NEAT1/miR-222-3p/CDKN1B in HG-induced mesangial cell hypertrophy. * indicates *P*<0.05, *** indicates *P*<0.001 compared with NG or IgG. ^###^ indicates *P*<0.001 compared with HG. Data were expressed as mean ± SD, and experiments were repeated three times.

Next, we investigated the upstream regulator of NEAT1 expression in mesangial cells. JAK/STAT signaling has been shown to be activated upon exposure to HG (Wang et al., 2002), so we tested if JAK/STAT is indeed involved in the transcription of NEAT1. We treated the cells exposed to HG with AG490, a selective JAK2/STAT3 inhibitor, and checked the expression of NEAT1. Indeed, the inhibitor reduced the expression of NEAT1 in time-dependent manner (Figure 6A), suggesting that JAK/STAT transcription factors induce the transcription of NEAT1. In addition, a reporter system using the NEAT1 promoter to express luciferase showed an increase in the luciferase activity upon HG exposure which was blunted by AG490 treatment (Figure 6B), further validating the transcriptional role of JAK/STAT in expressing NEAT1. We also scanned for the potential binding site of JAK/STAT in the promoter of NEAT1, and found STAT binding motif (Figure 6C). To test if STAT3 binds to the promoter of NEAT1, we performed ChIP. Indeed, the STAT3 was found to bind to the promoter of NEAT1, and the binding further increased upon exposure to HG (Figure 6D). Collectively, these results demonstrate that JAK2/STAT3 promote the transcription of NEAT1 upon HG exposure.

## Discussion

Despite research elucidating several proteins’ critical roles in HG-induced hypertrophy, complete pathways and specific mechanisms need to be uncovered for targeted therapeutic opportunities. Our work demonstrates lncRNA NEAT1’s clear and specific role in HG-induced hypertrophy through its direct interaction with miR-222-3p. Through this interaction, miR-222-3p has limited binding capacity with CDKN1B, leading to elevated expression and the well observed phenotype of CDK inhibitor-mediated hypertrophy and cell growth arrest. Additionally, we showed STAT3 pathway regulates the expression of NEAT1, which is activated through HG. This uncovers a clear pathway, from exposure to HG, which is common in diabetic milieu, to CDK inhibitor expression (Figure 6E), in which mesangial hypertrophy occurs, paving the way for future therapeutic research and development for the treatment of diabetes.

NEAT1 has been previously shown to be a player in mesangial cell cycle regulation, however specific regulators of NEAT1 remained unclear (Huang et al., 2019). In this study, we identified STAT3 as an upstream regulator of NEAT1 expression. STAT3 is activated not only by HG but also by many inflammatory cytokines (Huynh and Chand, 2019). JAK/STAT signaling plays an important role in the pathogenesis of diabetic nephropathy (Chuang and He, 2010; Matsui and Meldrum, 2012; Brosius and He, 2015), and this pathway has been shown to promote proliferation in renal glomerular mesangial cells (Wang et al., 2002). Furthermore, STAT3 is overexpressed in the kidney of diabetic nephropathy patients compared to the ones from normal patients (Zhang et al., 2017). Activation of JAK/STAT has been shown to induce the expression of TGF-β1, collagen IV and fibronectin which causes fibrosis and leads to glomerulosclerosis in diabetic nephrophathy (Wang et al., 2002). In this study, we identified NEAT1 as an additional factor that is directly induced by STAT3 in transcriptional level, and is functionally important in regulating hypertrophy in mesangial cells. This finding highlights the novel function of STAT3 in the pathogenesis of diabetic nephropathy by regulating hypertrophy in addition to promoting inflammation and fibrosis.

It would also be interesting to test if STAT3 regulates the expression of NEAT1 in the context of immune regulation, and if miR-222-3p mediated regulation would be physiologically important in this context as well. A recent study has shown that NEAT1 promotes cell proliferation, migration and metastasis in breast cancer by inhibiting miR-146b-5p (Li et al., 2020). Our study has identified miR-222-3p as a novel target of NEAT that has important functional role in regulating hypertrophy. These examples of ceRNA regulation by lncRNA invite future research for similar regulation by other noncoding RNAs.

In addition to uncovering NEAT1’s role in hypertrophy, we also uncovered an important role of miR-222-3p in regulating HG-induced hypertrophy. Previous work on miR-222-3p has emphasized its role in a variety of cancers, but none have elucidated its role in diabetic hypertrophy (Takigawa et al., 2016; Chen and Li, 2020). This once again underscores the diversity of RNA, from lncRNA to miRNA, and their crucial role in complex gene expression regulation.

CDKN1B expression has been shown to be regulated both in transcriptional level as well as in protein stability (Wolf, 2000). TGF-β induces the expression of CDKN1B in the transcriptional level (Liu and Preisig, 1999). HG induced expression of CDKN1B is also dependent on protein kinase C (PKC) (Wolf et al., 1997). Furthermore, mitogen activated protein (MAP)-kinases are shown to phosphorylate CDKN1B and increase its stability (Wolf, 2000). All these factors have been shown to be important in regulating renal hypertrophy and contribute to the pathogenesis of diabetic nephropathy (Wolf, 2000). This study further identifies a new mechanism through which NEAT1 modulates the expression of CDKN1B in the protein translation level by sequestering miR-222-3p that targets CDKN1B mRNA.

Our study uncovered an important role of NEAT1 lncRNA in regulating cell hypertrophy. We propose a complete pathway in which HG leads to the activation of STAT3, resulting in NEAT1 regulation of the miR-222-3p/CDKN1B axis. These results lead to a new opportunity for novel agents that may inhibit or modulate any of the individual players, enabling more specific treatment of HG-induced hypertrophy.

## Data Availability Statement

The original contributions presented in the study are included in the article/Supplementary Material, further inquiries can be directed to the corresponding authors.

## Author Contributions

LL, JC and CZ performed the experiment. YG, WL, and JH performed the data analysis. WL, LD, and ZL designed the study. LL and JL prepared the manuscript. All authors contributed to the article and approved the submitted version.

## Funding

This work was funded by Summit Discipline of Clinical Traditional Chinese Medicine in Pudong New Area of Shanghai (PDZY-2018-0601) and National Natural Science Foundation of China (82074261).

## Conflict of Interest

The authors declare that the research was conducted in the absence of any commercial or financial relationships that could be construed as a potential conflict of interest.
